# Draft genome sequence of *Mycobacterium intracellulare* strain HYG9370 isolated from Japanese Black Cattle in Japan

**DOI:** 10.1128/mra.00946-24

**Published:** 2024-12-10

**Authors:** Reiko Kawaguchi, Yuichi Ueno, Kanako Kurita, Toshiyuki Wako, Yasuyuki Mori, Yohsuke Ogawa

**Affiliations:** 1 Himeji Livestock Hygiene Service Center, Himeji, Hyogo, Japan; 2 National Institute of Animal Health, National Agriculture and Food Research Organization, Tsukuba, Ibaraki, Japan; 3 Institute of Crop Sciences, National Agriculture and Food Research Organization, Tsukuba, Ibaraki, Japan; 4 ZEN-NOH Institute of Animal Health, National Federation of Agricultural Cooperative Associations, Sakura, Chiba, Japan; 5 Sapporo Research Station, National Institute of Animal Health, National Agriculture and Food Research Organization, Sapporo, Hokkaido, Japan; University of Strathclyde, Glasgow, United Kingdom

**Keywords:** *Mycobacterium*, cattle

## Abstract

Here, we report the draft genome sequence of *Mycobacterium intracellulare* strain HYG9370 (ID: JD563) isolated from a clinically healthy Japanese Black steer. The genome information of this strain provides valuable insights into the genetic diversity of *M. intracellulare*, which has been isolated from multiple hosts and environments.

## ANNOUNCEMENT

*Mycobacterium intracellulare* is a causative agent of non-tuberculosis mycobacterial diseases in humans. It has been isolated from the environment and livestock, promoting molecular epidemiological analyses to understand its genetic diversity and trace the sources of human infection (1–3). In cattle, *M. intracellulare* can cause false positive results in Johne’s disease antibody tests (4), making it an important bacterium for livestock hygiene. Although over 100 genome sequences of *M. intracellulare* are available in the National Center for Biotechnology Information (NCBI) database, none are from bovine-derived strains.

Herein, we report the draft genome sequence of *M. intracellulare* strain HYG9370 (ID: JD563) isolated from a 1-year-old, clinically healthy Japanese Black beef steer in Hyogo Prefecture, Japan (35°4′N, 134°8′E), in 2008. For bacterial isolation, 1 g of feces appropriately collected from the rectum under the supervision of Himeji Livestock Hygiene Service Center (Diagnostic No. 1175), was suspended in 20 mL of sterile saline. After vigorously shaking followed by settling, the surface fluid was collected and processed with 0.75% hexadecylpyridinium chloride before pelleting and resuspension in an antibiotic solution (50 µg/mL each of vancomycin, nalidixic acid, and amphotericin B) as detailed previously (5). The sample was cultivated for 3 months on a commercial agar slant for *M. avium* subsp. *paratuberculosis* (Kyoritsu Seiyaku, Japan) at 37°C under aerobic conditions. A single colony was restreaked and cultured on Middlebrook 7H10 agar (Becton Dickinson, USA) supplemented with 10% BBL Middlebrook OADC Enrichment (Becton Dickinson), 7.5% egg yolk, 2 µg/mL of mycobactin (Kyoritsu Seiyaku), 0.5% glycerol, and 50 µg/mL of amphotericin B (5) at 37°C under aerobic conditions (strain HYG9370).

Genomic DNA was extracted using a Johne-Pure-Spin kit (FASMAC, Japan) with modifications that included adding 1 mg/mL RNase (NIPPON GENE, Japan) and agitating for 8 s using MicroSmash MS-100 (Tomy Seiko, Japan) (6). A DNA library was prepared using the TruSeq Nano DNA Low-throughput Library Preparation kit (Illumina, USA). DNA was sheared into approximately 450 bp fragments using a LE220 ultrasonicator (Covaris, USA). DNA fragments were tagged using TruSeq DNA Single Indexes (Illumina). The fragment size and concentration were measured using a 2100 Bioanalyzer (Agilent, USA). Sequencing was performed on an Illumina MiSeq platform with 300 bp paired-end reads using MiSeq reagent kit v3 (Illumina).

In total, 1,451,060 raw reads (436,769,060 nt) were processed using Platanus_trim version 1.0.7 (http://platanus.bio.titech.ac.jp/pltanus_trim) with the default parameters. Assembly was conducted using unicycler ver. 0.5.0 with the default settings (7), yielding 58 contigs totaling 5,391,780 nt, with an *N*_50_ of 264,850 nt and a GC content of 68.06%. The average coverage was 69.2×. Genome annotation was performed using the NCBI Prokaryotic Genome Annotation Pipeline ver. 6.8 (8). One of the original 58 contigs was identified as contaminated and removed, resulting in the draft genome containing 57 contigs, 4,958 coding genes, 1 rRNA, and 46 tRNAs. Analysis using CheckM version 1.2.3 showed 96.08% completeness and 2.98% contamination. Strain identification using JSpeciesWS (https://jspecies.ribohost.com/jspeciesws/) (9) confirmed the strain as *M. intracellulare*, showing a 96.96% average nucleotide identity with *M. intracellulare* strain TMC 1406^T^ (accession number CP076382.2).

## Data Availability

The draft genome sequence of *M. intracellulare* strain HYG9370 is available under accession number GCA_041504425.1. Raw sequence data are available under SRA accession number DRX565817, BioProject PRJDB18580, and BioSample SAMD00805255.
